# Dog olfactory receptor gene expression profiling using samples derived from nasal epithelium brushing

**DOI:** 10.1186/s40575-022-00116-7

**Published:** 2022-05-20

**Authors:** Naoual Azzouzi, Anne-Sophie Guillory, Gilles Chaudieu, Francis Galibert

**Affiliations:** 1 UMR6290 IGDR (Institut de Génétique Et Développement de Rennes), Université de Rennes 1, CNRS, 35000 Rennes, France; 2 Clinique Vétérinaire Pole Santé Chanturgue, 63100 Clermont-Ferrand, France

## Abstract

Dogs have an exquisite sense of olfaction. In many instances this ability has been utilized by humans for a wide range of important situations including detecting explosives and illegal drugs. It is accepted that some breeds have better senses of smell than others. Dogs can detect many volatile compounds at extremely low concentrations in air. To achieve such high levels of detection, the canine olfactory system is both complex and highly developed requiring a high density of olfactory receptors capable of detecting volatiles. Consequently the dog genome encodes a large number of olfactory receptor (OR) genes. However, it remains unclear as to what extent are all of these OR genes expressed on the cell surface.

To facilitate such studies, a nasal brushing method was developed to recover dog nasal epithelial cell samples from which total RNA could be extracted and used to prepare high quality cDNA libraries. After capture by hybridization with an extensive set of oligonucleotides, the level of expression of each transcript was measured following next generation sequencing (NGS). The reproducibility of this sampling approach was checked by analyzing replicate samples from the same animal (up to 6 per each naris). The quality of the hybridization capture was also checked by analyzing two DNA libraries; this offered an advantage over RNA libraries by having an equal presence for each gene. Finally, we compared this brushing method performed on living dogs to a nasal epithelium biopsy approach applied to two euthanized terminally ill dogs, following consent from their owners.

Comparison the expression levels of each transcript indicate that the ratios of expression between the highest and the least expressed OR in each sample are greater than 10,000 (paralog variation). Furthermore, it was clear that a number of OR genes are not expressed.

The method developed and described here will allow researchers to further address whether variations observed in the OR transcriptome relate to dog ‘life experiences’ and whether any differences observed between samples are dog-specific or breed-specific.

**Supplementary Information:**

The online version contains supplementary material available at 10.1186/s40575-022-00116-7.

## Lay summary abstract

All animals living in the wild depend on their sense of smell (olfaction) to find food, sexual partners and escape to predators. Domestic dogs are descendants of wolves and consequently have an acute and highly developed sense of smell. In addition many dog breeds have been subjected to strong selection practice by humans, with the aim of increasing their ability to perform a number of tasks highly dependent upon an acute sense of smell.

As in most other mammals, dogs are equipped with a large number of olfactory receptors (OR) expressed on cells lining the surface of the olfactory sensory epithelium in their nasal airways. Olfactory receptors are able to detect volatile molecules in the air to register a discreet smell sensed in the brain. To date, only one published study has compared the level of expression of dog OR genes to that seen in a small sample of other mammals. Furthermore this study only collected samples from three deceased dogs of mixed breed. A major obstacle to conducting such studies has been developing a less invasive and simple methods for sampling canine nasal epithelial samples.

In this paper we describe a non-aggressive method to recover olfactory sensory neuron samples from living animals. This method will now enable further analysis of dog OR RNA profiling of many dogs and breeds, allowing us to document their variation and determine if this is influenced by environmental or following life experiences. We present data to validate this method and summarize some preliminary findings.

## Introduction

Olfactory receptors were discovered 30 years ago by Buck and Axel, who identified a novel sub-family of GPCR proteins (G protein coupled receptor) in rat olfactory epithelium [1]. The importance of this discovery, which transformed the field of olfaction, was subsequently recognized by their award of the Nobel Prize for physiology or medicine in 2004. For all wild animals, olfaction is a vital function. It participates critically in the foraging of food, the selection of sexual partners, prevention from danger and predation. In humans, even if these functions are usually fulfilled differently, olfaction remains an important sense and people suffering from anosmia or even hyposmia are at a disadvantage.

Since, the discovery of several OR gene transcripts in rat olfactory epithelial tissue, many other animal specie’s olfactory gene repertoires have been identified by using full genome DNA sequencing. Such studies show that these genes represent the largest gene family in the genome with several hundred members scattered across multiple chromosomes [2–7].

However, there is a paucity of studies relating to gene transcription and expression to decipher the number of expressed genes in olfactory tissues [8–13] These studies showed that 90% of human and rat and 70% of dog OR genes are expressed, although at widely different levels and with no or limited differences between males and females. These studies also showed that gene transcription is not limited to intact OR genes but that some pseudogenes are also expressed [14, 15]. It is widely recognized that olfaction is an extremely important and well-developed sense in dogs. However, what makes some dogs more than others excel at detecting drugs, explosives or even diseases in humans remains unknown [16, 17]. Many dog breeds, such as hunting dogs, have been selectively bred to have an acute sense of olfaction. Comparative studies across different dog breeds thus provides an important opportunity to investigate the relationship between olfactory gene expression and polymorphism and their functional consequences. Following characterization of the dog genome sequence [18], we determined the dog OR repertoire [15, 19] and identified genetic polymorphism in a number of OR genes within a cohort of 48 dogs; these dogs represented six different breeds recognized for their different olfactory capabilities [20, 21]. The number of OR genes present and the extent of their genetic diversity are important parameters in determining olfactory capabilities in all mammals. However, variation in ORs expression levels, as well as of the proteins implicated in the odorant transduction signal toward the brain, are also important in explaining individual differences; at present this is not characterized or fully understood. Analysis of the dog olfactory epithelium has been limited due to a number of challenges; these include the ethical and painless tissue sampling, anatomical issues given the large size of the olfactory epithelium (which can be up to 200 cm^2^ for an adult German Shepherd) [22].

Two ways of accessing olfactory epithelial tissue currently exist. The first is by collecting samples from euthanized incurable dogs or from deceased dogs following an accident. In principle, sampling nasal epithelium following euthanasia gives access to the whole olfactory epithelium (OE). However, relying on euthanasia only presents limitations; these include the number of samples able to be collected, the range of dog breeds investigated and the circumstances relating each situation. Restriction of nasal epithelium sampling to just instances of euthanasia will seriously limit research aimed at characterizing the full variation of olfactory transcriptomes, including the effects of breed, training and the age.

A second sampling option is possible through gentle scraping of the nasal epithelium with the aid of a nasopharyngeal swab, similar to those used at the hospitals in human Otorhinolaryngology (ORL) services. We have pioneered and investigated the use of such an approach as a means of developing an ethical and minimally invasive general method of OR sampling which can be utilized in many situations.

## Material and methods

### Olfactory epithelium samples

**Fig. 1:**
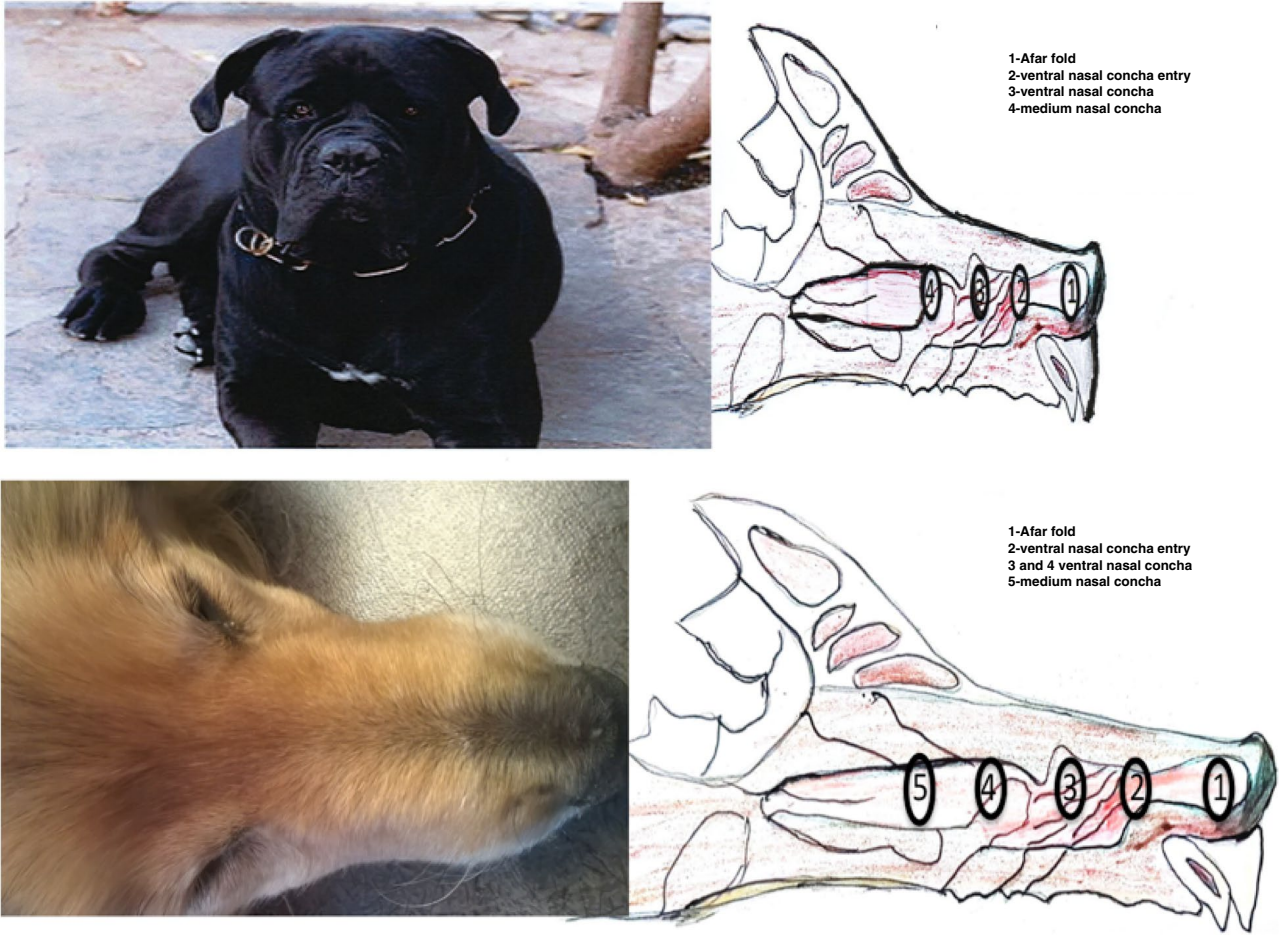
Schematic view of the sample localizations. from the Cane Corso and Golden Retriever euthanized dogs**.** Samples made by the brushing approach correspond to position 3. In addition, biopsy samples made from the Cane Corso and the Golden Retriever euthanized dogs correspond to positions 1 to 4 or 1 to 5 respectively

All samples of nasal epithelium were collected at the Clinique Vétérinaire (Pole Santé Chanturgue-63100 Clermont-Ferrand –France) under general anesthesia (Ketamine-Imalgene 1000®and xylazine—Rompun®—aa, 0.1 mL/kg IV, Isoflurane-O2). These were performed for chirurgical purposes by gentle brushing made with endo-cervical DOC cyto-brushes (Medispo.com). Two of the samples collected, one from a Bichon and a second from a Golden Retriever, were used for total mRNA sequencing. Multiple samples were also collected from four dogs (a Belgian Shepherd, a West Highland WhiteTerrier, a Whippet and a Labrador Retriever) (Fig. 1).

All animals included were present for programmed surgical interventions and their samples were collected with owner consent and ethic committee approval. After recovery of the nasal epithelium, brushes were immediately placed into a tube containing 1.5 ml of RNA later solution. These were immediately sent to the laboratory where they were stored at – 80C° until subsequent nucleic acid extraction.

Several biopsies were also taken with owner consent from two euthanized dogs; a Cane Corso and a Golden Retriever that were at the terminal phase of cancer. These biopsies were taken as a means of sampling the olfactory epithelium at different locations. These samples were processed as described above.

### Nucleic acid isolation

Total RNA was extracted and purified with Nucleospin RNA kits (Macherey Nagel). Following titration with a Nanodrop spectrometer, the quality and purity of the RNA samples were assessed by use of a BioAnalyzer (Agilent 2100). Only RNA samples with a RIN score of ≥ 8 were used for analysis. DNA extraction from the Golden Retriever and Cane Corso samples were made using Nucleospin tissue kits.

### Sample processing

The Bichon and Golden RNA samples were sent to the French Genomic Platform (Centre INRA Toulouse- Midi Pyrénées) for library construction and NGS analysis. RNA extracted from other samples were sent to Integragen.

### Library construction

cDNA libraries were constructed as follows: 1 µg of total RNA, polyA RNA molecules were purified using poly-T oligo attached to magnetic beads. The attached RNA molecules were fragmented using divalent cations under elevated temperature to obtain approximately 300nt pieces. This was followed by library construction and addition of Illumina adapters. For each of the two DNA libraries, 600 ng of genomic DNA were fragmented by sonication and purified to yield fragments of 150–200 bp. Paired-end adaptor oligonucleotides from the NEB kit were then ligated followed by eight PCR cycles.

### Hybridization capture

Following amplification of the cDNA and DNA libraries, 120 ng of each were hybridized to the SureSelect oligoprobe capture set, made up of 45,142 overlapping biotinylated oligonucleotides (120 mers), covering the complete OR open reading frames. Oligonucleotides were designed and synthesized by Agilent [23] from the sequences of 999 OR genes and pseudogenes and 19 control gene sequences identified in Canfam3.1 [24].

### Sequence data analysis

Sequencing was performed on a HIseq 4000 sequencer (Illumina, 2 × 125nt) using the v4 chemistry/HBS Hiseq kit. One line was used for each sample to produce up to 300 × 10^6^ reads per sample. Image analysis and base calling were performed using Illumina Real-Time Analysis software version 2.7.3 with default parameters. Raw sequence data produced by the Genotoul platform and Integragen were sent to the laboratory for processing and analysis. The two sequence extremities were trimmed to remove the remaining primer sequences and any bases with poor quality base calling often present at the extremities of the reads with Cutadapt [25]. Trimmed sequences were then aligned using STAR.v2 through Galaxy (Sigenae) [25]. The resulting BAM files were analyzed with Samtool, Bedtool, Cufflink and Stringtie [26] using the Toulouse Genocluster and with the Geneious suite [27]. Statistical analysis of the data and Heatmap constructions based on the FPKM (Fragments Per Kilobase Million, i.e. the number of sequence reads mapping on a given gene) values were made using the Manhattan and Ward method (using R language) by ‘in house’ written lines of command [28].

## Results

### OSN transcriptome analysis

Samples of nasal epithelium tissues were obtained from a male Bichon and a female Golden Retriever, both aged of 8 years. As the olfactory neurons might be contaminated by other cell types (thus reducing the level of neuron-specific transcripts and the OR transcripts) each sample was deep sequenced and up to 300 million reads obtained. This maximized the chances of capturing transcript differences from poorly expressed genes. This was in contrast to the 60 million reads previously reported for murine olfactory neurons [10]. As shown in Table 1, approximately 90% of the reads could be mapped to unique positions. This high percentage of mapping reflected the good quality of the RNA, libraries and the sequencing itself. Consequently, we are confident that the two Figs. 0.27 and 0.31, representing the percent mismatch between sequence reads aligned with the reference genome (CanFam3.1), are both indicative and representative of any differences due to polymorphism.

**Table 1:** Summary of RNA-Seq data

	**Read number**	**Read length**	**Uniquely mapped %**	**Mismatch %**
Golden Retriever	299,595,502	263	88.79	0.27
Bichon	291,999,387	245	89.99	0.31
Murine OSN [10]	58,234,129	Not documented	59.28	Not documented

The percentage of mismatch noted for the Bichon and Golden Retriever samples were obtained by comparison of their sequences to that of the reference dog genome (CanFam 3.1 Ensembl.org 2011). The percentage of uniquely mapped genes were calculated with STAR.v2 [24]

Analysis of the sequence data allowed us to identify many genes and calculate their respective FPKM values (i.e. the number of reads corresponding to each transcript, a metric defining the abundance of all transcripts (Additional file 1a, b) and OR transcripts (Additional file 2). For these two samples, despite their deep sequencing only 14% and 16% of the OR gene transcripts were detected with an FPKM > 0.1. This corresponded to 112 Bichon and 104 Golden Retriever OR genes respectively, in contrast to 90% for the murine and human OR repertoires and 70% for canine OR repertoire [10, 13].

As summarized in Table 2, 88% and 90% of all the annotated genes (ENSEMBL.org) and 62% and 56% of the non-annotated genes are expressed at a detectable level. These high percentages of expressed genes are probably due to the multi-cellular composition of the samples. Based on respective FPKM values, the 10 most expressed identified genes are listed in Table 3. A comparison between dog gene expression and their murine orthologues [10], indicates strong differences. None of the highly expressed dog genes was found to be strongly expressed in the murine tissue. SCGB1A1, the most highly expressed gene in the Bichon sample, is not detected in a murine sample; VMO1 at the second position in the two dog samples ranks at position 1498 in the mouse sample; the same applies to TAGNL2 at position 6750. Similarly, the olfactory major protein (OMP), a protein characteristic of the olfactory tissues, and the third most expressed transcript in murine OSN, ranks at positions 2502 and 1796 in the two dog samples (Table 4). The Gα sub unit of the G(olf) protein encoded by *GNAL,* a key protein in the transduction pathway, ranks at position 9 in the mouse sample and 10,680 and 8767 in the two dog samples analyzed. The large differences in gene ranking observed between the dog and murine samples, strongly suggests that in the dog samples analyzed, the olfactory sensory neurons (OSN) were contaminated by other cellular types, such as the supporting tissue cells. This would dilute the expression of the OSN genes and comparatively increase that of non-OSN genes [29, 30].

**Table 2:** Percentage of known and unknown genes. The known genes are genes identified and named in the ENSEMBL.org database. The unknown genes correspond to transcripts covering sequences with the characteristics of coding genes but not identified as such yet and quoted “novel gene” in the database. FPKM (Fragments Per Kilobase of Exon per Million of Fragments Mapped) were calculated with GENEIOUS [26]

	**Known genes**	**% of genes with FPKM > 0.1**	**Unknown** **genes**	**% of unknown genes with FPKM > 0.1**	**Highest FPKM**
Bichon	14.540	88	13.159	62	62.980
Golden Retriever	14.554	90	16.230	56	52.340

**Table 3:** The 10 most highly transcribed genes. Genes ranking at the 10 first positions but present in one sample only are underlined

BICHON			GOLDEN RETRIEVER		
Gene Name	FPKM	Description	Gene Names	FPKM	Description
SCGB1A1	62,98	Secretoglobin family 1A member 1	TFF1	52,34	trefoil factor 1
VMO1	56,515	vitelline membrane outer layer 1 homolog	VMO1	47,833	vitelline membrane outer layer 1 homolog
TAGLN2	41,031	Transgelin 2	ENSCAFG00000030140	46,288	Novel gene
ENSCAFG00000009876	39,431	Novel gene	ENSCAFG00000009876	42,706	Novel gene
BPIFA1	39,131	BPI fold containing family A member	B2M	31,104	Beta-2-microglobulin
GSTM4	23,355	GLUTATHIONE S TRANSFERASE MU 4	TAGLN2	26,382	Transgelin 2
GSTM3	22,523	GLUTATHIONE S TRANSFERASE MU 3	CSTB	24,205	Cystatin B
CYP2A13	18,820,5	Cytochrome P450 family 2 subfamily A polypeptide 13	GAPDH	23,532	Glyceraldehyde-3-phosphate dehydrogenase
UBB	15,585	Ubiquitin B	FTH1	22,835	Ferritin heavy chain Ferritin heavy chain, N-terminally processed
GAPDH	15,574	Glyceraldehyde-3-phosphate dehydrogenase	GPX2	21,69	Glutathione peroxidase 2

**Table 4:** Level of expression of a couple of key OSN transcripts. This table shows that the ratio of the level of expression between the most expressed OR and the α subunit of the Golf (GNAL) differs according to the species. In the case

	**OMP/ Rank**	**OMP/FPKM**	**Highest OR/Rank**	**Highest OR/FPKM**	**GNAL/Rank**	**GNAL/FPKM**
Bichon	1,796	173	8,767	10	4,415	50
Golden Retriever	2,502	152	10,680	10	4,167	68
Murine OSN	3	1,185	9	1,072	380	89
Murine OE	16	1,370	55	399	1,427	35

## OR Targeted transcriptome analysis

### Reliability and reproducibility of the approach

**Fig. 2:**
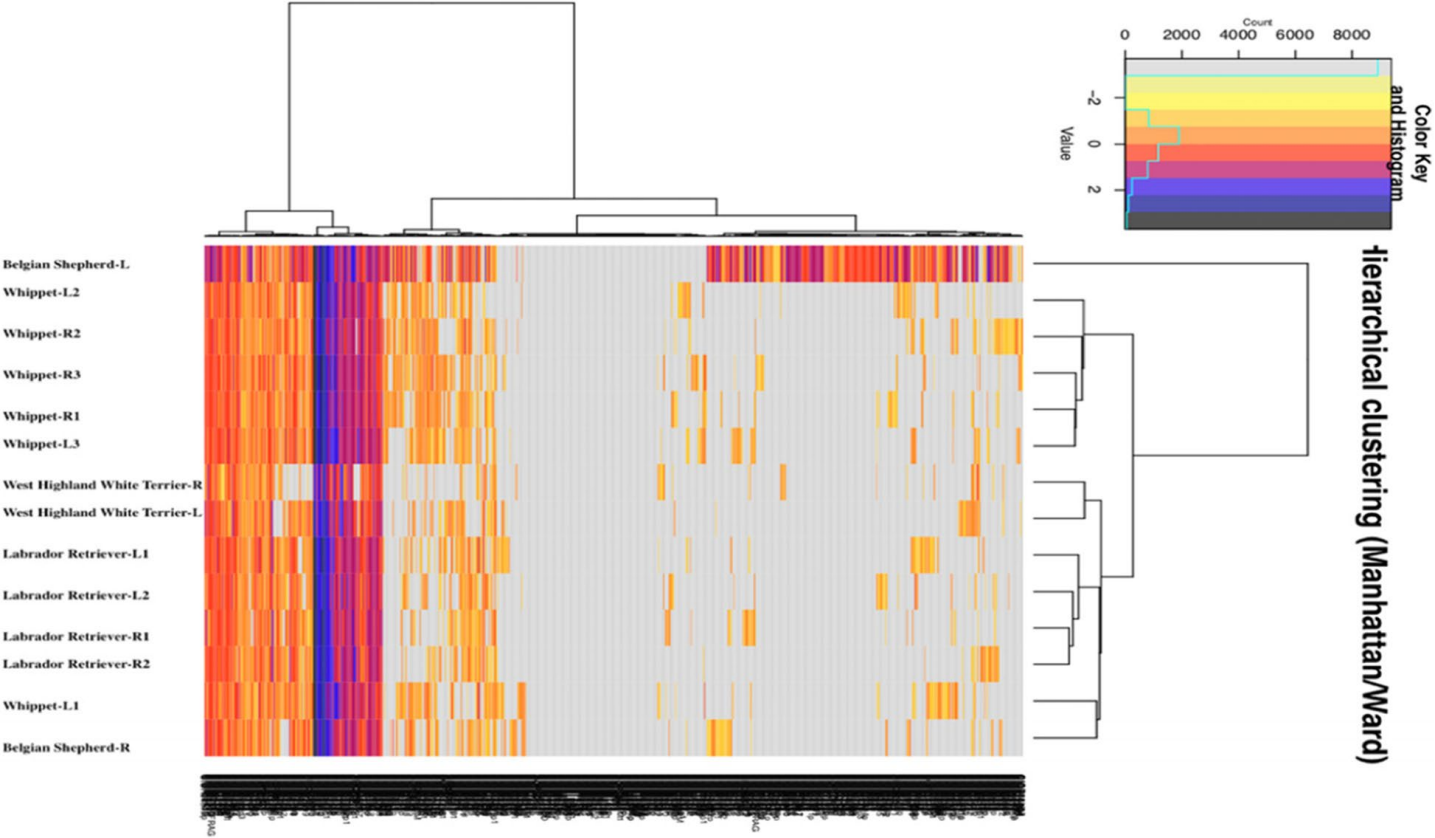
Multiple samples clustering. Hierarchical clustering constructed with all OR FPKM log values, that correspond to the number of times each transcript was sequenced, which itself depend on their concentration in the libraries (Additional file 3). Most of the samples from the same animal are in the same cluster, the main exception are the two Belgian Shepherd samples, one of them being clustered with the Labrador Retriever samples, the second being alone. This overall good clustering indicates the good reproducibility of the sampling and of the analysis. The distribution of the two Belgian Shepperd samples in two different clusters suggests a pathological problem [30]

Fourteen samples from four dogs (i.e. two to six per dog), were analyzed to appreciate the reproducibility of the sampling method and the differences that might exist between the right and left nostrils. As shown (Additional file 3) a strong correlation exists between the different FPKM values obtained for the same dog, regardless of whether the right and left nostril samples are compared or the different samples from the same nostril are compared. In the heat map presented in Fig. 2 hierarchical clustering is seen for the four Labrador Retriever samples, for the two West Highland White Terrier samples and for four out of the five Whippet samples. A discrepancy was seen for the two Belgian Shepherd samples which are not grouped. This suggests a sampling or a sequencing problem. Alternatively, a particular physiological condition could have induced a different transcription profile of one of the two nostrils of this dog [31]. Nevertheless, a coherent grouping for the majority of the samples indicated good reproducibility of the sampling itself and the good quality of the sequencing. Moreover, the grouping of the different samples of each dog indicated little difference between the right and left nostrils.

### Hybridization capture effects

To appreciate whether the hybridization capture process might have biased the FPKM values of the RNA transcripts, two DNA libraries, made from two dog samples, a Cane Corso and a Golden Retriever were analyzed. As each gene is present in two copies in any sample, the FPKM values of each pair of ortholog genes (i.e. the same gene in the Cane Corso and the Golden Retriever) should be identical or highly similar. The FPKM values obtained for the different OR genes are shown in Additional file 4. As shown the reproducibility of the capture is excellent with a correlation of 0.972 as calculated with the Pearson test.

However, not all OR genes of the same sample were captured with the same efficiency [32] (Additional file 4): CfOR1812 and CfOR0039p genes, for example, were efficiently captured leading to 1363 and 1357 FPKM values respectively. In contrast, the capture of CfOR0183 and CfOR0783p genes led to much lower FPKM values of 83.4 and 95.5 respectively. Since each gene is present in two copies, these differences in FPKM values between different paralogue OR genes are mostly due to differences in oligonucleotide hybridization efficiency, which are dependent upon the sequences themselves [32]. To obtain a more realistic view of the OR transcriptome, the FPKM values of the RNA transcripts were normalized (Additional file 5). This was done by conditioning each crude RNA FPKM value (Additional file 3) by a factor corresponding to the ratio of the FPKM values of this gene, as obtained by the analysis of the two DNA gene libraries.

One of the key observations of the data summarized in Additional file 5, is the large extended range of expression regardless of the samples used. Values ranged from above several thousand FPKM for the most expressed OR down to 0.1 for the least expressed. These data indicate that a variable, but large, proportion of the OR genes are not even detectable, having (if transcribed) an FPKM value below 0.1. If one concentrates on the OR genes having an FPKM value ≥ 1% of the most expressed OR gene of the sample, then these results are even more surprising. About 30 genes are above this limit, as already observed for the Bichon and Golden Retriever of which their data were not normalized and for which we obtained 39 and 37 OR genes above this limit.

To address the issue of whether the normalization made was appropriate and correct we compared 20 of the most expressed Bichon and Golden OR genes (Additional file 1a and b) with that the 20 most expressed OR genes of the West Highland White terrier, Whippet and Labrador (Additional file 5). This revealed that 9 out of these 20 genes are present in all samples, indicating that the correction made was correct.

A further consideration was whether the low proportion of OR genes being expressed reflected reality or was a consequence of a capture effect and/or of the variable number of sequencing reads per sample.

**Fig. 3:**
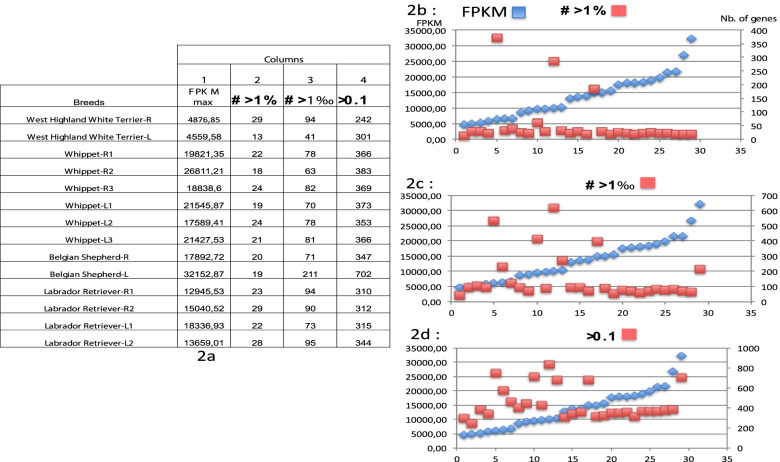
Absence of correlation between the FPKM values and the number of expressed OR. This figure is made of four Tables (2a to 2D). Table 2a for each sample, the FPKM values of the most highly expressed OR (column 1). In column 2 are the number of OR expressed at a FPKM value ≥ to 1%. Column 3 indicates the number of OR expressed with an FPKM value ≥ to 1^0^/_00_ and column 4 gives the total number of detected OR (FPKM ≥ 0.1). To check whether the FPKM values to which the highest expressed OR are detected impact the number of detected OR, we compared the number of detected OR (column 2, 3 or 4) to that of the highest FPKM values [1]. As shown in plots 2b, c and d there is no correlation between the highest FPKM values and the number of expressed OR genes indicating that the low number of detected OR is a reality and might be a characteristic of dogs

To address this, the DNA FPKM values were plotted for the Cane Corso and Golden Retriever samples in blue and the FPKM values of the corresponding RNA in red (Additional file 6a and b). As shown, no correlation existed between the DNA and RNA FPKM values for any genes: e.g. Golden Retriever CfOR 12F06 or CfOR 0268 genes. Furthermore, a large number of genes were not transcribed at a detectable level, whereas their cognate genes were well captured by the same set of oligonucleotides. Thus, the fact that a large number of OR transcripts (660 Golden Retriever and 742 Cane Corso) was not detected cannot be due to the failure of the hybridization capture. Whereas the number of reads could have impacted the number of expressed OR genes, the comparison of the ratios and the plots detailed in Fig. 3 (a-d), indicated no correlation between the FPKM values and the number of expressed OR genes. Similarly, the number of OR genes having an FPKM value ≥ 0.1 in the different Whippet samples is not affected by the FPKM value of the most highly expressed OR. Around 370 OR were detected in Whippet samples whereas the highest FPKM values varied between1,3249 for sample L1 and 21,545 for sample L2 (Additional file 5).

### Spatial segregation of OR expression

Spatial segregation of OR gene expression within the olfactory epithelium has already been documented although to what extent a similar situation exists in dogs was unclear. Thus, we considered whether the limited number of OR genes expressed in the samples analyzed was a consequence of this spatial segregation [19]. To approach address this biopsy samples were taken from a Cane Corso and a Golden Retriever respectively (Fig. 1). FPKM values of the different OR transcripts are provided in Additional file 7. These data files show that up to 414 and 512 OR genes are not expressed in the Cane Corso and Golden Retriever samples respectively.

## Discussion

The dog genome contains a large number of OR genes [4]. This explains in part, the wide variety of volatile components a dog can recognize and how in a complex environment it can recognize an odor to which it has been trained. To be effective the OR genes have to be transcribed and expressed. Of these two aspects, we presently know little. Several transcriptome studies have analyzed the spectrum of OR genes expressed in humans, rats, and mouse [8–12] but until recent, few studies have focused on dogs [13]. A major reason for this has been the difficulty of collecting appropriate samples in an ethical and practical way.

As shown with the Bichon and the Golden retriever samples collected, the gentle brushing of the nasal epithelium allows us to recover sufficient olfactory neurons to extract their total RNA content and to perform a transcriptomic analysis. However, the relative quantification of the specific transcripts of the olfactory neurons such as those of the OMP or the Gα subunit of the G(olf) protein, indicates that these two samples were heavily contaminated by other cells such as the supporting cells [29, 30]. The main consequence of such contamination is a dilution of the OSN transcripts. Thus, the OMP mRNA transcript, the third most expressed transcript in murine OSN [10], ranks at positions 2502 and 1796 in the dog samples and many of the OR transcripts are even not detected in spite of applying deep sequencing analysis.

This contamination problem of dog samples obtained by brushing limits their transcriptome analysis. Firstly, it decreases the accuracy with which the FPKM values are obtained, secondly, it limits the number of detected genes and thirdly this approach is expensive in terms of sequencing costs. To circumvent these problems, two possibilities are envisaged. The first one relates to a purification step of the OSN fraction by immunoprecipitation with a specific antibody. The second strategy, which we prefer for its simplicity relys on hybridization and capture of the sequences of interest with a set of oligonucleotides. A method often used to enrich a fraction in a given set of nucleotide sequences.

In another series of analyses, prior to sequencing, cDNA libraries were subjected to a capture of the entire sequences of all the canine OR genes by hybridization with a large panel of long and overlapping oligonucleotides. To check the reproducibility and efficiency of the capture process, we prepared two DNA libraries made from samples taken from a Cane Corso and a Golden Retriever. Using the same set of oligonucleotides, we captured the OR gene nucleotide sequences and then sequenced them. As each gene is present in two copies, we anticipated that (a) the FPKM values of each gene should be similar or identical in the two libraries and (b) the comparison of the FPKM of the different OR genes within each library will reveal the efficiency of the capture method itself.

In Additional file 4, the calculated FPKM values are plotted; in blue for the Cane Corso sample and in red for the Golden Retriever. As shown, most of the blue and red dots are concordant or extremely close, indicating good reproducibility of the capture itself, and supported by a Pearson correlation test.

However as seen in Additional file 4, comparison of the FPKM of the different OR genes within each of the two sample indicates a large difference, confirming the previous observation that the efficiency of hybridization capture is in part sequence dependent [32]. This dependency does not prevent comparison between samples and the tight clustering (11 out of 14 samples) of the captured sequences based on their FPKM values indicates a good reproducibility of the entire process i.e. from the recovery of the samples up to the sequencing itself (Fig. 2). Within the limits of the techniques used, this grouping also indicated no major differences between the level of expression of the mRNA extracted from left and right naris of the same animal.

To compare the transcription profile of the different paralogue OR transcript within a sample, a correction factor for crude FPKM values should be applied. Since each gene is present as two copies in any genome, the FPKM ratio of the two OR genes (Additional file 4) of a pair is a consequence of the efficacy difference of the capture itself for each of these two genes. As explained in the result section (Additional file 5), a correction was applied to each crude RNA FPKM value by a factor corresponding to the ratio of the FPKM values of this gene to the most expressed gene of the sample calculated from the DNA gene libraries.

Previous studies have been made regarding the transcriptome analysis of several mammal olfactory tissues [8–13] These studies have shown that nearly all OR genes would be expressed at a detectable level. In contrast to this, in our study, it appears that far less dog OR dog gene transcripts are detected. Interestingly, no more than 30 genes reach an FPKM value ≥ 1% of the OR the most expressed in the sample (Additional file 5). As shown in Fig. 3, we observed no correlation between the highest FPKM values in any sample and the number of expressed OR genes in the corresponding sample and in all samples, we observed a very large range of expression as much as 10,000 times.

The absence of a correlation between the FPKM values of the DNA genes and the RNA transcripts, indicates the low number of expressed genes is not a consequence of a failure of the hybridization capture but might correspond to a characteristic of the canine RNA olfactory profile, in at least of the samples analyzed (Additional file 6). Consequently we considered whether the relatively low number of OR genes being expressed could be due to the sampling itself, as a consequence of a strong regionalization of the expression of the different OR genes all along the canine olfactory epithelium, as previously observed with rat [19]. To tackle this question, we prepared several samples representing different site locations of a Cane Corso and Golden Retriever olfactory epithelium. As shown in Additional file 7, although the RNA profiles of the different biopsies are not strictly identical in either of the two animals, importantly up to 512 and 414 OR genes (i.e. 56 and 46% of the whole set of OR genes) are not detectable whatever the samples and their localization was in the OE. Furthermore up to 40% are silent when one combines the data of the 10 biopsy samples. At present we have no explanation regarding the much larger number of expressed OR genes found by Saraiva et al. who reported that only 14% of OR genes were not detected [13]. This could be due to breed differences sampled, (in the case of Saraiva et al. it was a mixed breed). Nevertheless, it is important to keep in mind, that the absolute number of observed expressed OR genes, is probably less important in characterizing the RNA profiling of any species than the range to which the genes are expressed. It is unclear what meaning in biological terms such low expression of genes may represent. Whatever the issue of the absolute number of dog OR genes expressed, it is very important to consider the observation that the ratio of expression of the human and mouse OR genes RNA is much lower [8, 12] to that found by Saraiva [13] and from our study reported here.

## Conclusion

The focus of this study was aimed at evaluating a non-invasive sampling approach which could be ethically and practically used in research applications study the olfactome of dogs across a wide range of different variables including breed diversity, age, behavioral conditioning and environmental situations.

Our approach, was inspired by human otorhinolaryngology routine practice and the need to establish a veterinarian-led procedure which is easy to establish and does not inflict pain or impact on animal wellbeing. The data we present here support that this is a valid and robust qualitative and quantitative approach to investigating expression profiles and its variation. The different samples obtained by nasal brushing and biopsy are highly similar to each other. Up to approximately half of dog OR genes appear to be silent or not detectable although this needs to be established in sufficient sample sizes across a wide range of breeds. There is also a significant number of genes where their expression is either very low or exceptionally high. The absolute number of genes expressed genes may not necessarily represent an important biological parameter given the low level of expression of some genes. A concerted international effort is now required to investigate and characterize the dog olfactome and determine is impact on canine health and welfare.

## Supplementary Information

**Additional file 1.** Bichon and Golden Retriever Gene profiling.

**Additional file 2.** Bichon and Golden Retriever OR genes profiling.

**Additional file 3.** OR FPKM results of 14 independant cDNA libraries.

**Additional file 4.** DNA hybridization capture.

**Additional file 5.** Normalized transcript FPKM data of the 14 samples.

**Additional file 6.** The capture effect.

**Additional file 7.** OR biopsy results, normalized FPKM values.

## Data Availability

All data will be freely available upon publication. Any further request could be obtained from the corresponding author galibert@univ-rennes1.fr.

## Abbreviations

OR: Olfactory Receptor
NGS: Next Generation Sequencing
GPCR G: Protein Coupled Receptor
OE: Olfactory eEpithelium
Bp: Base pair
FPKM: Fragment Per Kilobase Million
OMP: Olfactory Major Protein
OSN: Olfactory Sensory Neuron

## References

[1] Buck L, Axel R (1991). A novel multigene family may encode odorant receptors: a molecular basis for odor recognition. Cell..

[2] Glusman G, Yanai I, Rubin L, Lancet D (2001). The complete human olfactory subgenome. Genome Res.

[3] Godfrey PA, Malnic B, Buck L (2004). The mouse olfactory receptor gene family Proc. Natl Acad Sci USA.

[4] Quignon P, Giraud M, Rimbault M, Lavigne P, Tacher S, Morin E, Rtout E, Valin AS, Lindblad-Toh K, Nicolas J, Galibert F (2005). The dog and rat olfactory receptor repertoires. Genome Biol..

[5] Montague MJ, Gang L, Gandolfi B, Khan R, Aken BL, Searle SMJ, Minx P (2014). Comparative analysis of the domestic cat genome reveals genetic signatures underlying feline biology and domestication. Proc Natl Acad Sci USA.

[6] Niimura Y, Matsui A, Touhara K (2014). Extreme expansion of the olfactory receptor gene repertoire in African elephants and evolutionary dynamics of orthologous gene groups in 13 placental mammals. Benome Res.

[7] Azzouzi N, Barloy-Hubler F, Galibert F (2014). Inventory of the cichlid olfactory receptor gene repertoires: identification of olfactory genes with more than one coding exon. BMC Genomics..

[8] Rimbault M, Robin S, Vaysse A, Galibert F (2009). RNA profiles of rat olfactory epithelia: individual and age-related variations. BMC Genomics.

[9] Ibarra-Soria X, Levitin MO, Saraiva LR, Logan DW (2014). The Olfactory Transcriptomes of Mice. PLoS Genet.

[10] Kanageswaran N, Demond M, Nagel M, Schreiner BSP, Baumgart S (2015). Deep Sequencing of the Murine Olfactory Receptor Neuron Transcriptome. PLoS One.

[11] Wang Z, Zhou Y, Luo Y, Zhang J, Zhai Y, Yang D, Zhang Z, Li Y, Storm DR (2015). Gene Expression Profiles of Main Olfactory Epithelium in Adenylyl Cyclase 3 Knockout Mice. Int J Mol Sci.

[12] Olender T, Keydar I, Pinto JM, Tatarskyy P, Alkelai A, Chien MS (2016). The human olfactory transcriptome. BMC Genomics.

[13] Saraiva LR, Riveros-McKay F, Mezzavilla M, Abou-Moussa EH, Arayata CJ (2019). transcriptomic atlas of mammalian olfactory mucosae reveals an evolutionary influence on food odor detection in humans. Sci Adv..

[14] Crowe ML, Perry BN, Connerton JF (1996). Olfactory Receptor-Encoding Genes and Pseudogenes Are Expressed in Humans. Gene.

[15] Prieto-Godino LL (2016). Peraro MD, Benton R. Olfactory receptor pseudo-pseudogenes. Nature.

[16] Campbell LF, Farmery L, Creighton-George SM, Farrant PBJ (2013). Canine Olfactory Detection of Malignant Melanoma BMJ Case Rep.

[17] Willis CM, Britton LE, Swindells MA, Jones EM, Kemp AE, Muirhead NL (2016). Invasive melanoma in vivo can be distinguished from basal cell carcinoma, benign naevi and healthy skin by canine olfaction: a proof of principle study of differential volatile organic compound emission. Br J Dermatol.

[18] Lindblad-Toh K, Wade CM, Mikkelsen TS, Karlsson EK, Jaffe DB, Kamal M (2005). Genome sequence, comparative analysis and haplotype structure of the domestic dog. Nature.

[19] Quignon P, Kikness E, Cadieu E, Touleimat N, Guyon R, Renier C (2003). Comparison of the canine and human olfactory receptor gene repertoires. Genome Biol.

[20] Tacher S, Quignon P, Rimbault M, Dreano S, Andre C, Galibert F (2005). Olfactory receptor sequence polymorphism within and between breeds of dogs. J Hered.

[21] Robin S, Tacher S, Rimbault M, Vaysse A, Dréano S, André A, Hitte C, Galibert F (2009). BMC Genomics.

[22] Quignon P, Galibert F. Chapter 4. In: Jezierski T, Ensminger J, Papet LE, editors. Canine Olfaction Science and Law: advances inforensic science, medicine, conservation, and environmental remediation. Boca Raton: CRC press, Cop.; 2016. p. 39-48.

[23] Gnirke A, Melnikov A, Maguire J, Rogov P, LeProust EM (2009). Solution hybrid selection with ultra-long oligonucleotides for massively parallel targeted sequencing. Nat Biotechnol.

[24] CanFam3.1/Ensembl/release-101/hisat2/Canis_lupus_familiaris.CanFam3.1.6

[25] Dobin A, Davis CA, Schlesinger F, Drenkow J, Zaleski C, Jha S (2013). STAR: ultrafast universal RNA-seq aligner. Bioinformatics.

[26] Li H, Handsaker B, Wysoker A, Fennell T, Ruan J, Homer N, Marth G (2009). The Sequence Alignment/Map format and SAMtools. Bioinformatics.

[27] Kearse M, Moir R, Wilson A, Stones-Havas S, Cheung M, Sturrock S (2012). Geneious Basic: An integrated and extendable desktop software platform for the organization and analysis of sequence data. Bioinformatics.

[28] Strauss T, von Maltitz MJ (2017). Generalising Ward’s Method for Use with Manhattan Distances. Plos One.

[29] Farbman AL, Brunjes PC, Rentfro L, Michas J, Ritz S (1988). The effect of unilateral l naris occlusion on cell dynamics in the developing rat olfactory epithelium. J Neurosci.

[30] Kavoi B, Makanya A, Hassanali J, Carlsson HE, Kiama S (2010). Comparative functional structure of the olfactory mucosa in the domestic dog and sheep Ann Anat.

[31] Coppola DM, Waggener CT (2012). The effects of unilateral naris occlusion on gene expression profiles in mouse olfactory mucosa. J Mol Neurosci.

[32] Hoppman-Chaney N, Peterson LM, Klee EW, Middha S, Courteau LK, Ferber MJ (2010). Evaluation of Oligonucleotide Sequence Capture Arrays and Comparison of Next-Generation Sequencing Platforms for Use in Molecular Diagnostics. Clin Chem.

